# Introducing a vignette experiment to study mechanisms of ethnic discrimination on the housing market

**DOI:** 10.1371/journal.pone.0276698

**Published:** 2022-10-27

**Authors:** Abel Ghekiere, Pieter-Paul Verhaeghe, Stijn Baert, Eva Derous, Stijn Schelfhout

**Affiliations:** 1 Department of Sociology, Vrije Universiteit Brussel, Brussels, Belgium; 2 Department of Economics, Ghent University, Ghent, Belgium; 3 Department of Sociology, Ghent University, Ghent, Belgium; 4 Department of Work, Organisation and Society, Ghent University, Ghent, Belgium; University of Georgia, UNITED STATES

## Abstract

Ethnic discrimination on the housing market has been subject of research for years. While a field experimental approach is widespread, alternative attempts to objectively measure mechanisms of discrimination on the housing market are scarce. In line with labor market research, we stress that to reduce rental discrimination against ethnic minorities, we need understanding its underlying mechanisms. This is the first paper that introduces a vignette experiment to do so. We distinguish between four mechanisms put forward in the literature but hardly ever empirically tested: agent taste-based discrimination, owner taste-based discrimination, neighborhood taste-based discrimination and statistical discrimination, in a multifactorial vignette experiment among 576 pre graduate real estate student. In addition, our experimental design allows us to examine whether unequal treatment is heterogeneous by property owner and neighborhood characteristics.

## 1. Introduction

Ethnic discrimination on the housing market has been subject of research for years [1, 2]. While recent review articles have shown that these rates have been declining over time in both the United States [2, 3] and Europe [4], there still exists a large amount of research that provides evidence that discrimination in the search for housing is still a prominent problem for ethnic minorities, both in cities as in rural areas [1, 4]. More concretely, a recent meta-analysis of [1] found that rental candidates belonging to the ethnic majority group are still almost twice as likely to be chosen by landlords and real estate agents than candidates belonging to the ethnic minority group. Although measuring ethnic discrimination with large scale field experiments is a well-established practice, less is known about the underlying mechanisms and motivations for discriminatory behavior [5, 6]. In line with research on the labor market [7–9] we stress that to reduce ethnic discrimination on the rental market, we need understanding its underlying mechanisms.

The current two dominant mechanisms in the economic literature on discrimination are statistical and taste-based discrimination. From an economic point of view, real estate agents will treat ethnic minorities unfavorably because of either their distaste towards a member of that group (taste-based discrimination; [10]) or the use of aggregated information from the group to judge the applicant in the situation of too little individual information (statistical discrimination; [11]). Even though these theories are at the core of mostly all economic research on discrimination [12], they lack a certain specificity when it comes to understanding the reasoning of the discriminating real estate agent.

First, the binary economic view on mechanisms of discrimination is restricting theorizing on drivers of discrimination on the housing market. Results from a recent literature review on mechanisms of discrimination on the labor market by Lippens [13] pointed out that due to the lack of consistent evidence of the two dominant economic mechanisms it is hard to argue that discrimination can be fully explained by either one. Attempts to add nuance to these mechanisms on the housing market such as Combes and colleagues’ “neighbor discrimination” or Verstraete and Verhaeghe’s “owner taste-based mechanism” add complexity to the drivers of rental discrimination and should be elaborated further [14, 15].

Second, the two dominant economic mechanisms, which are mainly adopted from labor market research, are likely to be different in specific housing market contexts. This specificity mainly relates to the tightness of the rental market, in which the economic cost of discriminating (as described in Becker’s taste-based discrimination mechanism; [10]) could be close to zero due to the high demand and low supply for housing. In the original formulation by Becker, hiring discrimination is called “economical irrational” because it decreases the productivity and thus the profit of a firm. However, in tight rental markets, not inviting an ethnic minority candidate for a viewing would doubtfully affect the profit of the agent or affect the chance to rent out the dwelling because the realtor would easily find other good rental candidates [15]. Additionally, the role of the real estate agent as a gatekeeper and mediator between client and applicant is unique and implies a specific approach to the mechanisms behind discrimination and selection [16, 17]. They are regarded as the intermediate link between unique clients and candidates, which can result in a tension between clients’ requests and the real estate agent’s behavior.

Finally, different client- or neighborhood characteristics could mediate forms of discrimination [18–20] and therefore could imply variation in mechanisms in the decision-making process of the real estate agent. In the original economic mechanisms, taste-based and statistical discrimination, there is little room for variation through differences in context even though they do allow for forms of variation in the personal drivers for distaste or observed group differences [10, 21, 22].

The goal of this paper is threefold. First, we specify the mechanisms behind ethnic discrimination by real estate agents. We add nuance to the scattered literature on mechanisms of discrimination by including two additional mechanisms of taste-based discrimination in our theoretical framework, the ‘owner taste-based’ and ‘neighborhood tasted-based’ mechanism. In total, four mechanisms are presented in the theoretical framework. Second, the distinction between these mechanisms is tested in a lab experiment among pre-graduate students in real estate and related study subjects in Belgium. By including different forms of reasoning for selection in a multifactorial vignette experiment, we are able to assess which mechanisms are dominant in the process of screening applicants in the rental process. Third, we examine how different discriminatory requests by clients affect the invitation rates and drivers for selection by real estate agents. Research shows that real estate agents are very willing to act upon discriminatory request [15, 23] however different requests could lead to different behavior. By altering between different types of request we can assess the underlying motivations and dominant mechanisms of each one.

Besides its theoretical contribution, the current study has a clear methodological relevance. While field experiments on the housing market are widespread [1], they lack the ability to disentangle the mechanisms that are prior to discriminatory behavior [5]. This article is the first to introduce a multifactorial vignette experiment on the rental housing market that attempts to counter these shortcomings. Considering that this is the first study with a multifactorial survey experiment on housing market discrimination.

This article proceeds as follows. First, we elaborate more on the four mechanisms of discrimination. Subsequently, we explain the logic of the multifactorial vignette experiment and apply it on the context of rental discrimination with its four mechanisms. In the data and methods section, we explain how we have carried out this new vignette design among Belgian students in real estate and related study topics and how we will analyze the data. After that section, we present the findings of our structural equation models and regression analyses. In the final section, we draw a few conclusions and reflect about the implications for further research.

## 2. Mechanisms of discrimination

In this study, we distinguish between four different mechanisms of ethnic discrimination on the housing market: agent taste-based discrimination, owner taste-based discrimination, neighborhood taste-based discrimination and statistical discrimination.

The first mechanism, agent taste-based discrimination, has its roots in Becker’s *Economics of discrimination* [10]. Becker argues that discrimination is motivated by exogeneous preferences (tastes) towards certain groups of people we interact with. The agent’s own prejudice leads the realtor to discriminate in order not to work with these ethnic minority groups. This behavior could lead to costs for the agent, being the loss of a potential rental candidate for a dwelling that is under the realtor’s supervision. This cost is taken into account by the agent which contributes to the argument that this form of behavior is a conscious and intentional act [14, 24].

In contrast, owner taste-based discrimination is defined as the act of discriminating because of the explicit prejudices of the client, the owner of a property [15, 25]. This mechanism occurs when a client explicitly asks the real estate agent not to rent out their property to ethnic minorities, a question that arises often in the Belgian housing market. Engaging in this request is defined as an intention to discriminate [24], it is prior to the act of discriminating and therefore cannot be labeled as discriminatory behavior. Although this mechanism is most salient when working with a client that has an explicit discriminatory request, it could also occur when the client has less explicit requests. The real estate agent could make the assumption that the client would favor a majority candidate over a minority candidate through certain perceived prejudices about the client. This mechanism is considered to be similar to the customer taste-based mechanism of discrimination on the labor market [10].

Third, the mechanism of neighborhood taste-based discrimination refers to the act of discriminating because of the explicit or perceived prejudices of the building or neighborhood the dwelling is situated in. Recent studies that include contextual factors in their analysis have shown more discrimination in poorer, more ethnic diverse neighborhoods [19, 26, 27]. In the study by Krysan and colleagues [28] it became clear that neighborhoods with mostly black residents were portrayed as less favorable than those that were mixed or mostly white, residential preference is shaped not only by class-based characteristics but also by racial composition [28]. Yinger [25] hypothesizes that: “*rental agents for all-white apartment buildings discriminate against blacks because the entry of a few blacks could lead to the exit of many or all white tenants and hence to extensive turnover costs*. *Real estate brokers cultivate contacts in their community to attract potential buyers and sellers*. *In a prejudiced white community*, *therefore*, *brokers discriminate against blacks to avoid alienating most of their potential clients*, *namely white residents*.*”* This mechanism is considered to be similar to the employee taste-based mechanism of discrimination on the labor market [10].

A final theoretical perspective describes discriminatory behavior as statistical [11, 29]. This mechanism of discrimination is based on ascribing group characteristics to an individual because of imperfect information on the individual. The agent uses the (perceived) average group value of characteristics to judge the ability of the applicant to rent out a dwelling. For example, the perceived financial situation of an ethnic group could be used to assess a candidate’s financial reliability [30]. These group values could result in inducing higher risks when working with ethnic minorities because of the assumptions that they have fewer resources. However, statistical discrimination could equally arise when the assessment of the financial reliability of an individual candidate is muddled by skepticism about self-reported income. This assessment could reflect an agent’s experience with ethnic minority customers, but it cannot, of course, reflect the qualification of customers. In any case, acting on this belief is a form of statistical discrimination, because it uses a preconceived characteristic on average as a signal about an individual candidate. Finally, we argue that statistical discrimination could also arise from a believe about the probability of a successful transaction in relation to the rental price. As Ondrich [6] found, increasing rental price leads to a decreasing believe of a successful transaction with an ethnic minority candidate.

Previous studies concerning the dominant mechanism of ethnic discrimination are inconclusive. On the one hand [31], and [18] found evidence for taste-based discrimination being the dominant mechanism. On the other hand, the meta-analysis of Auspurg [4] found strong evidence for statistical discrimination in rental housing markets. We will assess this ongoing debate by including these mechanisms in a multifactorial survey experiment, elaborated in the section below.

## 3. Data

### 3.1 Multifactorial vignette experiment

To study the empirical value of these mechanisms, we employ a multi-factorial survey experiment. This experimental approach to surveys presents respondents with simulated representations of objects or conditions (vignettes), which vary according to certain features (dimensions) of various levels (variable values). In academic studies, such hypothetical explanations of cases and situations tested by respondents are becoming more and more popular [32]. The use of multi-factorial survey experiments to study discrimination has proven to be successful in numerous studies on labor market discrimination [33–36]. Due to the combination of experimental design features, such as randomization in the vignettes, with the advantages of heterogeneous respondent samples, the vignette experiment has become a well-established tool in social sciences [32, 37, 38]. However, we must take a few things into account. First, vignette experiments may still be prone to socially desirable response behaviour as the results of previous studies are rather inconclusive on this matter [39, 40]. Second, that by measuring discriminatory behavior with a multifactorial survey experiment, we measure the intentions to discriminate rather than the actual behavior as measured with field experiments.

Previous research used vignette experiments to study discrimination on mainly the labor market (e.g., [36, 41, 42]). This study is the first to investigate the four mechanisms to the context of the housing market with the use of a multifactorial vignette experience.

### 3.2 Participant selection

We conducted our experiment in September 2020 with students across different educational institutions in the Dutch-speaking part of Belgium–that offer real estate related programs. Real estate agents in Belgium are regulated professionals, which means that the agent is obliged to be accredited by the vocational institute of real estate before he/she is allowed to work as a realtor. The trajectory to become accredited includes getting a degree in a real estate related domain, finishing an internship with an accredited agent and passing a centralized proficiency test. The most common way of getting the degree is studying at a tertiary educational institution. In the Dutch-speaking part of Belgium, six higher educational institutions provide programs that include courses for aspiring real estate agents. All six institutions agreed to our request to conduct a vignette experiment among their students in programs that were real estate related. Eventually, we were able to conduct the experiment with 643 students ranging from first year to last year students, with a response rate of 91 percent. The experiment was a compulsory part of the student curriculum; hence we encountered only little selection effects in our sample and the composition of our sample reflects the proportions at the population level. An additional 67 respondents did not agree with the informed consent or did not finish the survey. The sample consists of a first group that studies real estate and a second group of students similar in age and demography but not in the specific real estate program. However, this second group of respondents are students in a more general management program from which students can choose to specify in real estate in later phases. We provided the professors with a link to the survey that was distributed to the students who conducted the experiment in the first weeks of the academic year, Table 1 describes the characteristics of the respondents in this study. The final, cleaned sample (using a Full Information Maximum Likelihood-option which accounts for missing values) included 576 respondents. There are no significant differences in characteristics and results between the different educational institutions.

**Table 1:** Characteristics of the respondents in the vignette study (n = 576).

	N	%
Study track		
Real estate	323	56%
Management / law	253	44%
Gender		
Male	330	57%
Female	246	43%
Country of birth of the mother		
Belgium	487	85%
Other	89	15%
Age		
18–20	410	71%
21–23	131	22%
24 +	35	7%

### 3.3 The experiment

The situations and design of the vignette experiment were set to be as closely corresponding to real job situations. In line with the recommendations from the study of Sauer and colleagues [32] concerning the design of multi-factorial surveys, we used a running text, a rating scale and randomly presented vignettes in our study. The experiment was to be completed on a computer.

Before starting the survey, a written consent was required from the participant. The ethical committee of the Faculty of Political and Social Sciences of the University of Ghent believes that this study is a clear reflection on the ethical aspects associated with both qualitative and experimental research. The committee gave a positive advice to the application.

On the first page of the survey, participants were informed about their role as a real estate agent, who is responsible for inviting candidates for viewing of an apartment. In addition, we mentioned some requirements for adequately performing this task, such as being professional and sales oriented [42].

First, the participant was presented with a vignette from a client who wanted to rent out its apartment. We created two characteristics that could vary across the client’s vignettes; the price of the dwelling that was being rented out and the discriminating request of the client. This enables us to measure variation in mechanisms according to different discriminatory requests and rental price. More concretely, the respondent was presented with a client that rented out an apartment with a high or a low rental price and with a requests that could be: (i) we only want quiet, clean renters who don’t disturb the neighbors, (ii) our neighbor had bad experiences with ethnic minorities, we don’t want that happening (iii) We want to rent out the apartment quick, it is a bit shabby and is located in a rough neighborhood, that’s why it is so cheap (iv) we’ve had bad experiences with ethnic minorities, so we don’t want to rent out to them anymore. We include these variables based on previous research on the housing market that indicated that these context variables have an effect on ethnic discrimination by realtors (for rental price see [20, 26], for discriminatory requests, see [15]).

Second, four rental candidates were presented to the respondents. The task of the respondent (who takes the role of real estate agent) was to rate the four candidates according to the vignette of the client (i.e., the owner of the rental dwelling). The information on the candidates included the name, contact details, marital status, number of kids and/or pets, net monthly income and the amount of rent paid in the current dwelling. All four applicants were married, have no kids and no pets. The only information that varied over participants was the name of the candidate and the level of the income. Alternatively, the typically Belgian sounding (native) names “Thomas Boey” and “Michiel van den Berghe” or the typically North-African sounding name “Mohammed El Malahi” and “Youssef Loutfi” were assigned to the application and the income was set either high (€2100) or low (€1500). We chose these names based on previous correspondence studies in Belgium and popular names of ethnic groups in Flanders. By doing so we limit the probability of variation in denotation of racial groups through names [43–45]. Thus, the same four candidates were presented (in a random order) to all the respondents, the variation lies only in the client’s characteristics. All possible combinations of factors result in a vignette universe of 8 (2 client characteristics x 4 client’s preferences) vignettes. Ideally, we aimed to test each vignette at least 30 times to create the highest internal validity, each respondent was presented with one client with four applicants, which results in a required sample of 240 observations for our study.

The respondent had to comment, for every candidate, on different items which signal drivers or reasons behind the choice of the candidate. These items are presented in Table 2. A 7-point rating scale indicated the likeliness to agree to the statement, ranging from (1): I do not agree to this statement, to (7): I agree with this statement.

**Table 2:** Mean rental candidates’ scores with respect to different mechanisms of discrimination and the final decision on a 7-point rating scale (n = 576).

	Mean		Difference
	(1)	(2)	(3)
	Native candidate	North-African candidate	(2)–(1)
**A. Attitudes related to model of taste-based discrimination**			
Agent taste-based mechanism:			
“As a real estate agent, I will enjoy working with this candidate”	5.18	4.90	-0.28 ***
“I can trust this candidate”	4.72	4.53	-0.19 ***
Owner taste-based mechanism:			
“My clients will be happy with the candidate”	5.35	3.35	-2 ***
“My clients match this candidate”	5.14	3.28	-1.86 ***
Neighborhood taste-based mechanism:			
“The candidate fits the neighborhood well”	4.51	4.28	-0.23 ***
“The candidate will feel good in the neighborhood”	4.50	4.28	-0.22 ***
**B. Attitudes related to model of statistical discrimination**			
“The candidate could have difficulty to pay rent in time”.	5.04	5.02	-0.02 ***
“Communication with this candidate could be difficult.”	5.06	4.40	-0.66***
**C. Decision**			
“I will invite this candidate for a first viewing.”	5.61	4.98	-0.63***

*Notes*. T-tests are performed to test whether the differences presented in Column (3) are significantly different from 0.

* p < 0,05

** p < 0,01

*** p < 0,001

Ethnic inequalities in the first two items, “As a real estate agent, I will enjoy working with this candidate” and “I can trust this candidate”, signal agent taste-based discrimination, which measures the respondent’s preference towards engaging with the applicant, irrespective of the client’s characteristics or the applicants’ income levels. Next, ethnic inequalities in the items: “My client will be happy with the candidate” and “My client matches this candidate”, signal the owner taste-based discrimination mechanism which measures the impact of client characteristics on the perceived fit with the candidate. In other words, they grasp the perceived judgement of the client towards the applicant. Third, we measure the neighborhood taste-based mechanism through ethnic inequalities in the items: “The candidate fits the neighborhood well” and “The candidate will feel good in the neighborhood”. These items root for the perceived fit of the applicant in the neighborhood.

Ethnic inequalities in the next items signal statistical discrimination. The specification of the risk is based on income adequacy and communication skills. While multiple sorts of information could be used to measure statistical discrimination (criminal history, prior rental history, references…), we used income and communication because they are two legal selection criteria in Belgium, crucial information for the rental process and both serve as a proxy for a candidate’s socio-economic situation. Additionally, working with students, these sources of information are most realistic and enquire no or little experience in the field. The first item: “The candidate will have difficulty to pay rent in time” uses the perceived financial reliability of the candidate based on his income (available for the participant) and the perceived financial reliability based on group values rather than individual income. The second item: “Communication with this candidate could be difficult” signals the perceived communication with an applicant.

Eventually, the participant had to make a judgement into what extent he/she will invite the applicant to a viewing: “I will invite this candidate for a first viewing”.

We first present the descriptive results of the data in chapter 3.4. To measure unequal treatment based on ethnicity in responses to these items, we compare the results for the ethnic majority and ethnic minority candidate. Subsequently, we assess, with the help of a SEM analysis, if we can extrapolate these items to their theoretical constructs, the mechanisms of discrimination. Finally, in additional regression analysis, we measure the effect of the variables of the experiment, ethnicity, price, income, and discriminatory request, on the mechanisms of discrimination.

### 3.4 Data description

Table 2 describes the data collected in this study. More specifically, it shows the respondents’ attitudes and invitation rates towards the fictitious rental candidates on a 7-point rating scale, classified by the ethnicity of the assigned rental candidate. First, we found that the majority candidate is favored over the minority candidate on every item of the experiment. While the differences between the majority and minority applicant are slim with respect to the items covering the agent taste-based mechanism (e.g., an average score of 5.18 against 4.90 for the statement: *as a real estate agent*, *I will enjoy working with this candidate*), they get bigger with respect to the owner taste-based mechanism (e.g., an average score of 5.35 to 3.35 for the statement: *my clients will be happy with the candidate)*. All differences between the applicants in regard of the taste-based mechanism are statistically significant. The big differences between the majority and minority candidates for owner taste-based discrimination can be possibly explained by the explicit discriminatory request of some of the clients. Second, for the construct of statistical based discrimination we found similar results. Again, the ethnic majority candidate is favored over the ethnic minority candidate. Next, in line with our expectations we find that the rental candidate with a Belgian sounding name has significantly higher chances to be invited for a viewing than the candidate with a North-African sounding name. These results are a first indication of discrimination towards ethnic minority rental applicants.

### 3.5 Methods

For the further analysis of the data, we use structural equation modelling (SEM), using the *lavaan* package (version 0.6–7) in R [46]. The main interest of a SEM analysis is the theoretical constructs (mechanisms of discrimination), which are presented by latent factors. The relationships between these factors are represented by regression coefficients [47]. To identify the latent factors, we first perform a confirmatory factor analysis. Through this statistical method we assess whether the items measured in the experiment (see Table 2) are properly nested in larger latent constructs (mechanisms of discrimination). The fit between the items and their latent constructs are measured with goodness-of-fit indices. We use robust statistics like *χ*^2^ goodness-of-fit, Tucker Lewis Index (TLI), Comparative Fit Index (CFI), and Root Mean Square Error of Approximation (RMSEA). We consider values above 0.95 to be suggestive of a good fit for the two relative indices, TLI and CFI [48]. For the RMSEA, a value lower than 0.06 indicates an acceptable fit [48]. Subsequent to the factor analysis, we perform a SEM analysis, in which we measure the effect of the mechanisms on the overall invitation rate of a candidate. This allows us to assess the dominant mechanisms and their impact on the applicants’ chances of being invited for a viewing. These measures are universal for all candidates, unequal treatment between the ethnic majority and minority candidate is only measured in the additional analysis in 4.2., the results presented in 4.1. show only whether the vignette experiment measures what we wanted to measure.

## 4. Results

**Fig 1:**
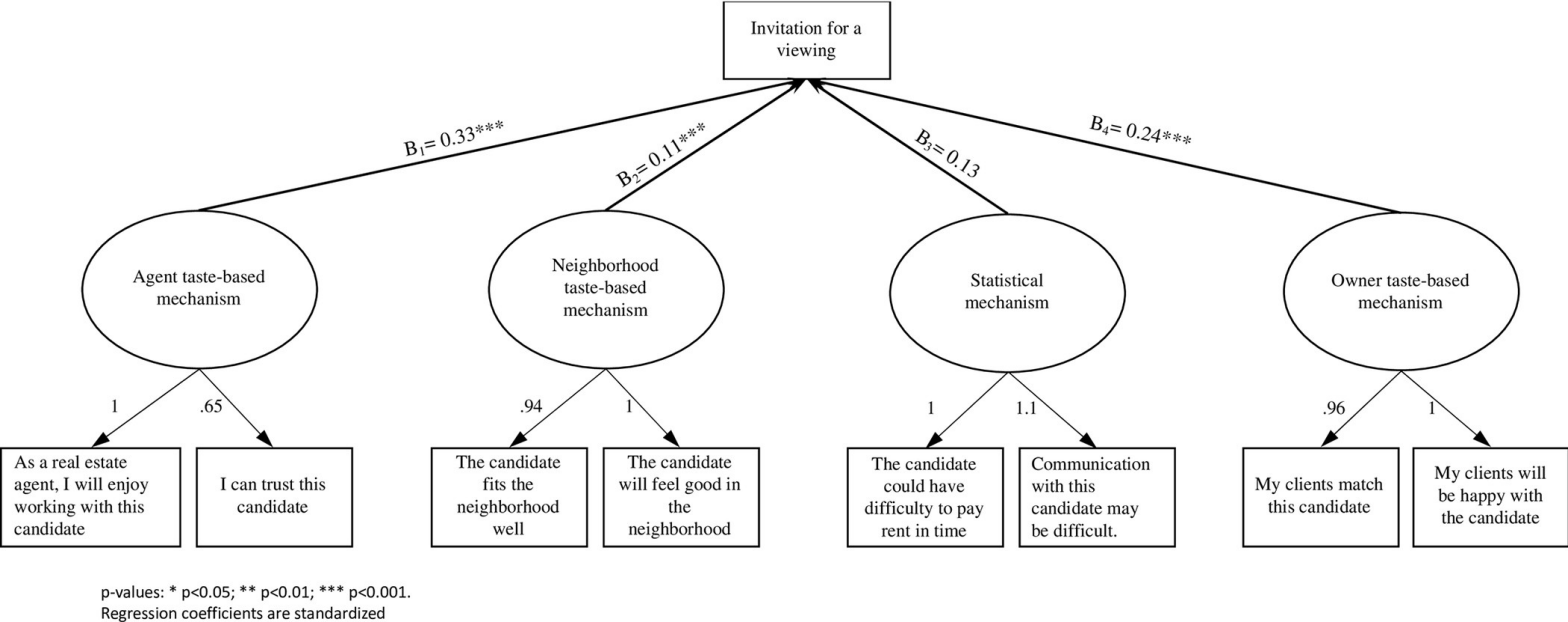
Structural equation model of the mechanisms of discrimination and the final decision (n = 576).

Fig 1 presents the CFA and SEM analysis of our study. First, the confirmative factor analysis shows that all four latent variables yield significant positive factor loadings ranging from 0.65 to 1.1. These factor loadings are correlation coefficients between the observed variables (items) and their latent constructs. The goodness of fit values indicates a very good fit between the model and the observed data, with Comparative fit index (CFI) = .99, the Tucker-Lewis fit index (TLI) = .98, and the RMSEA = .041. No post-hoc modifications were indicated from the analysis, and the residual analysis did not indicate any additional problems.

Following, the results of the Structural Equation Model show regression analyses that indicate the effects of the latent constructs on the invitation rate, presented in bold lines with standardized coefficients in Fig 1. It is noticeable that the statistical mechanism has no significant effect (b_3_ = 0,13) on the invitation of a candidate for a viewing. This implies that when controlled for the other mechanisms of selection, the perceived financial reliability and communicational strength of the applicant has no effect on the effective invitation of the applicant for a viewing. Second, the taste-based mechanisms do have a significant effect on invitation rates. The agent taste-based mechanism is the biggest actor in explaining the drivers behind the invitation rates (b_1_ = 0,33). In other words, the personal ‘connection’ between the respondents and the applicants is the main driver for selection of candidates. In addition, also neighborhood taste-based discrimination (b_2_ = 0,11) and owner taste-based discrimination (b_4_ = 0,24) appear to be important mechanisms.

Third, we ran the model separately for ethnic majority and ethnic minority candidates. This allows us to control whether the patterns are the same for the two subgroups. S1 and S2 Figs, show the SEM analysis for ethnic minority and majority candidates. We find that the model fits equally well in both analyses. The items are thus equally nested within their latent constructs. However, the regression analysis shows that the mechanisms only affect the invitation rates for the ethnic minority candidates. This implies that for the ethnic majority candidates, the invitation rate is independent of the selection mechanisms (at least for the ones that we measure). The decision, whether or not inviting a candidate for a viewing, shows no impact of personal or client related mechanisms. For the ethnic minority candidates, however, we did find significant effects of the mechanisms on the invitation rates, similar to those in Fig 1.

### 4.1 Additional analyses

To dig deeper in these mechanisms and to assess what the effect of different applicant and client characteristics are on the mechanisms, we perform additional linear regression analyses. We analyze the regression of the latent constructs, the combination of item responses, on the variables: ethnic origin of the rental candidate, income, rental price, and preference of the owner. The value of each latent construct is the weighted sum of their respective two items, defined in Fig 1. The results, as presented in Tables 3–7, show a clear impact of the variables that were included with experimental manipulation on the mechanisms behind ethnic discrimination. The following variables are used in the analysis:

*Ethnic origin* is a dichotomous variable that indicates whether the rental candidate is an ethnic majority or ethnic minority applicant.

*Income* is a dichotomous variable that indicates whether the candidate has either a high or a low income.

*Rental price* is a dichotomous variable that takes the value 1 for a high rental price, and 0 for a low rental price.

*Preference of the owner* is a categorical variable with four categories, that indicates the discriminatory request of the client. The reference category is the neutral request for clean and quiet renters.

**Table 3:** Linear regression analyses of the agent taste-based mechanism.

	Unstandardized (b) and standardized coefficients (β)				
	(1)	(2)	(3)	(4)	(5)
**Characteristics of the rental candidate**					
Ethnic origin	-0.282***	-0.171*	-0.247***	-0.309***	-0.242***
	(-0.149)	(-0.089)	(-0.129)	(-0.161)	(-0.127)
Income	0.358***	0.448***	0.365***	0.364***	0.364***
	(0.189)	(0.234)	(0.190)	(0.190)	(0.190)
**Rental price**	-0.01	0.023	-0.010	-0.10	-0.009
	(-0.005)	(0.012)	(-0.005)	(-0.005)	(-0.005)
**Preference of the owner** Ref.: clean and quiet renters					
Neighbor’s question	-0.146 **	-0.148*	-0.082	-0.147*	-0.147*
	(-0.068)	(-0.068)	(-0.038)	(-0.068)	(-0.068)
Scrappy neighborhood	-0.161 **	-0.165*	0.165*	-0.261*	-0.164*
	(-0.058)	(-0.059)	(-0.059)	(-0.093)	(-0.058)
Personal request	-0.249***	-0.256***	-0.256***	-0.256***	-0.187*
	(-0.120)	(-0.122)	(-0.122)	(-0.122)	(-0.089)
**Interaction terms**					
Ethnicity x Price	-	-0.065	-	-	-
		(-0.028)			
Ethnicity x Income	-	-0.167	-	-	-
		(-0.075)			
Ethnicity x Neighbor’s question	-	-	-0.131	-	-
			(-0.047)		
Ethnicity x Scrappy neighborhood	-	-	-	0.192	-
				(0.050)	
Ethnicity x Personal request	-	-	-	-	-0.136
					(-0.050)

*Note*: Standardized coefficient in parentheses. The variable ‘Preference of the owner’ is a categorical variable with “the request for clean and quiet renters” as the ref. category. Belgian is the ref. category for ethnic origin, low-income is the ref. category for income and low rental price is the ref. category for rental price. p-values

* p<0.05

** p<0.01

*** p<0.001.

**Table 4:** Linear regression analyses of the owner taste-based mechanism.

	Unstandardized (b) and standardized coefficients (β)				
	(1)	(2)	(3)	(4)	(5)
**Characteristics of the rental candidate**					
Ethnicity	-2.012***	-1.772***	-1.712***	-2.242***	-1.531***
	(-0.644)	(-0.568)	(-0.550)	(-0.721)	(-0.493)
Income	0.375***	0.480***	0.374***	0.371***	0.369***
	(0.120)	(0.154)	(0.120)	(0.119)	(0.119)
**Rental price**	-0.096	0.056	-0.094	-0.095	-0.095
	(-0.031)	(0.018)	(-0.030)	(-0.030)	(-0.030)
**Preference of the owner** Ref.: clean and quiet renters					
Neighbor’s question	-0.615***	-0.615***	-0.075	-0.615***	-0.615***
	(-0.175)	(-0.175	(-0.021)	(-0.176)	(-0.176)
Scrappy neighborhood	-0.105	-0.109	-0.110	-1.013***	-0.118
	(-0.023)	(-0.024)	(-0.024)	(-0.222)	(-0.026)
Personal request	-0.803***	-0.806***	-0.806***	-0.806***	-0.012
	(-0.234)	(-0.236)	(-0.236)	(-0.236)	(-0.004)
**Interaction terms**					
Ethnicity x Price	-	-0.300**	-	-	-
		(-0.079)			
Ethnicity x Income	-	-0.211*	-	-	-
		(-0.059)			
Ethnicity x Neighbor’s question	-	-	-1.080***	-	-
			(-0.041)		
Ethnicity x Scrappy neighborhood	-	-	-	1.797***	-
				(0.289)	
Ethnicity x Personal request	-	-	-	-	-1.584***
					(-0.362)

*Note*: Standardized coefficient in parentheses. The variable ‘Preference of the owner’ is a categorical variable with “the request for clean and quiet renters” as the reference category. Belgian is the ref. category for ethnic origin, low-income is the ref. category for income and low rental price is the ref. category for rental price. p-values

* p<0.05

** p<0.01

*** p<0.001.

**Table 5:** Linear regression analyses of the neighborhood taste-based mechanism.

	Unstandardized (b) and standardized coefficients (β)				
	(1)	(2)	(3)	(4)	(5)
**Characteristics of the rental candidate**					
Ethnicity	-0.227***	-0.192*	-0.094	-0.297***	-0.175**
	(-0.096)	(-0.082)	(-0.041)	(-0.127)	(-0.076)
Income	0.024	0.003	0.028	0.027	0.029
	(0.01)	(0.001)	(0.012)	(0.012)	(0.013)
**Rental price**	0.016	0.085	0.020	0.019	0.020
	(0.007)	(0.036)	(0.008)	(0.030)	(0.009)
**Preference of the owner** Ref.: clean and quiet renters					
Neighbor’s question	-0.252***	-0.249***	-0.013	-0.250***	-0.247***
	(-0.095)	(-0.095)	(-0.005)	(-0.095)	(-0.095)
Scrappy neighborhood	-0.428***	-0.421***	-0.420***	-0.699***	-0.417***
	(-0.124)	(-0.123)	(-0.123)	(-0.204)	(-0.123)
Personal request	-0.158 **	-0.157*	-0.157*	-0.158*	-0.078
	(-0.061)	(-0.062)	(-0.062)	(-0.062)	(-0.031)
**Interaction terms**					
Ethnicity x Price	-	-0.130	-	-	-
		(-0.046)			
Ethnicity x Income	-	0.050	-	-	-
		(0.018)			
Ethnicity x Neighbor’s question	-	-	-0.471***	-	-
			(-0.139)		
Ethnicity x Scrappy neighborhood	-	-	-	0.554***	-
				(0.119)	
Ethnicity x Personal request	-	-	-	-	-0.154
					(-0.047)

*Note*: Standardized coefficient in parentheses. The variable ‘Preference of the owner’ is a categorical variable with “the request for clean and quiet renters” as the reference category. Belgian is the ref. category for ethnic origin, low-income is the ref. category for income and low rental price is the ref. category for rental price. p-values

* p<0.05

** p<0.01

*** p<0.001.

**Table 6:** Linear regression analyses of the statistical based mechanism.

	Unstandardized (b) and standardized coefficients (β)				
	(1)	(2)	(3)	(4)	(5)
**Characteristics of the rental candidate**					
Ethnicity	-0.012	-0.031	-0.035*	-0.036*	-0.032
	(-0.02)	(-0.043)	(-0.049)	(-0.051)	(-0.051)
Income	0.386***	0.495***	0.492***	0.491***	0.491***
	(0.621)	(0.699)	(0.696)	(0695)	(0.695)
**Rental price**	-0.113***	-0.140***	-0.143***	-0.142***	-0.142***
	(-0.181)	(-0.196)	(-0.200)	(-0.200)	(-0.200)
**Preference of the owner** Ref.: clean and quiet renters					
Neighbor’s question	0.026	0.030	0.029	-0.030	0.030
	(0.037)	(0.037)	(0.036)	(0.037)	(0.037)
Scrappy neighborhood	0.015	0.011	0.011	0.003	0.011
	(0.016)	(0.011)	(0.011)	(0.003)	(0.011)
Personal request	0.033*	0.039*	0.039*	0.039*	0.042*
	(0.049)	(0.051)	(0.051)	(0.051)	(0.054)
**Interaction terms**					
Ethnicity x Price	-	-0.006	-	-	-
		(-0.007)			
Ethnicity x Income	-	-0.003	-	-	-
		(-0.003)			
Ethnicity x Neighbor’s question	-	-	0.001*	-	-
			(0.001)		
Ethnicity x Scrappy neighborhood	-	-	-	0.017	-
				(0.012)	
Ethnicity x Personal request	-	-	-	-	-0.006
					(-0.006)

*Note*: Standardized coefficient in parentheses. The variable ‘Preference of the owner’ is a categorical variable with “the request for clean and quiet renters” as the reference category. Belgian is the ref. category for ethnic origin, low-income is the ref. category for income and low rental price is the ref. category for rental price. p-values

* p<0.05

** p<0.01

*** p<0.001.

**Table 7:** Linear regression analyses of the invitation rates.

	Unstandardized (b) and standardized coefficients (β)				
	(1)	(2)	(3)	(4)	(5)
**Characteristics of the rental candidate**					
Ethnicity	-0.625***	-0.562***	-0.518***	-0.724***	-0.440***
	(-0.222)	(-0.200)	(-0.184)	(-0.258)	(-0.156)
Income	0.428***	0.455***	0.428***	0.428***	0.427***
	(0.152)	(0.162)	(0.152)	(0.152)	(0.152)
**Rental price**	-0.115	-0.074	-0.115	-0.115	-0.115
	(-0.040)	(-0.026)	(-0.040)	(-0.040)	(-0.040)
**Preference of the owner** Ref.: clean and quiet renters					
Neighbor’s question	-0.301***	-0.301***	-0.105	-0.301***	-0.301***
	(-0.095)	(-0.095)	(-0.033)	(-0.095)	(-0.095)
Scrappy neighborhood	0.044	0.044	0.011	-0.328**	0.044
	(0.011)	(0.011)	(0.011)	(-0.079)	(0.011)
Personal request	-0.403***	-0.403***	-0.403***	-0.403***	-0.089
	(-0.131)	(-0.131)	(-0.131)	(-0.131)	(-0.029)
**Interaction terms**					
Ethnicity x Price	-	-0.055	-	-	-
		(-0.017)			
Ethnicity x Income		-0.081	-	-	-
		(-0.024)			
Ethnicity x Neighbor’s question	-	-	-0.392**	-	-
			(-0.011)		
Ethnicity x Scrappy neighborhood	-	-	-	0.744***	-
				(0.132)	
Ethnicity x Personal request	-	-	-	-	-0.627***
					(-0.158)

*Note*: Standardized coefficient in parentheses. The variable ‘Preference of the owner’ is a categorical variable with “the request for clean and quiet renters” as the reference category. Belgian is the ref. category for ethnic origin, low-income is the ref. category for income and low rental price is the ref. category for rental price. p-values

* p<0.05

** p<0.01

*** p<0.001.

First, the results of the analysis in Table 3 show a negative effect from ethnicity on the agent taste-based mechanism (b = -0.282, *p* < 0.001), which implies a negative attitude or preference towards working together with an ethnic minority. A negative effect implies a worse assessment of the applicant, a positive effect implies a better assessment of the applicant. The agent taste-based mechanism is based on a purely personal preference towards a rental candidate and should not be influenced by other factors such as income, contextual factors or dwelling price [31]. This definition is supported by the non-significant interaction effects of both dwelling price and income with the ethnic background of the candidate. This result implies that ethnicity has a constant negative effect, not regarding the income of the applicant or the rental price which is proof for agent taste-based discrimination in our sample.

Second, the variation in discriminatory questions show that a personal request of the owner to exclude ethnic minorities (“*I would not like you to rent out to ethnic minorities”*) has the biggest (negative) impact on the respondent’s preference to work together with a candidate (β = -0.120, *p* < 0.001). Both the question related to the neighbor (“*My neighbor would not want me to rent out the dwelling to ethnic minorities”*) (β = -0.068, *p* < 0.01) and the comment related to the neighborhood the dwelling is situated in (“*It is a bit of a scrappy neighborhood*, *so we want to rent it out quick”*) (β = -0.058, *p* < 0.01), have negative effects but are smaller compared to the personal question.

Finally, when interacting these clients’ requests with the ethnicity of the candidate (3), (4), (5) we find no significant effects. The negative effect of the candidate’s ethnicity is constant and shows no influence from the interaction with income or owner’s preferences, which is again proof for agent-taste based discrimination.

From Table 4, it appears that the effect of ethnicity is most dominant for the owner taste-based mechanism. The ethnicity of the applicant has a substantial negative effect on the perceived fit between client and applicant (b = -2.012, p<0.001). As stated in the theoretical framework, the owner taste-based mechanism consists of items *the applicant fits the client well* and *My clients will be happy with the candidate*. The negative ethnicity effect is mostly explained by the discriminating questions that were asked by the client, from which the personal approach has the biggest effect (β = -0.362, p<0.001). From model 2 it appears that the rental price only has a significant effect for minority candidates, but not for natives: the perceived match between client and the North-African applicant is especially lower for more expensive dwellings, which means that in more expensive rental dwellings, the clients preference seems to be more important. Possibly because the price for not discriminating, and thus losing the client, is higher in these cases. In the same vein, also a high-income level of the minority candidate results in more owner taste-based discrimination. The question regarding the neighbor not wanting the client to rent out to ethnic minorities has a smaller significant negative effect (β = -0.041, p<0.001). Interestingly, when adding the interaction between the discriminatory request and ethnicity, the estimate for the majority candidate drops to almost zero. This indicates that the effect is almost totally ascribed to the ethnicity of the candidate, resulting in negative effects for the ethnic minority candidate. The customer’s request does not affect the respondent when judging the majority candidate in their ability to rent.

Subsequently, the neighborhood taste-based mechanism shows to be mostly affected by the neighbor’s discriminatory request (β = -0.138, p<0.001) (see Table 5). Interestingly, the applicant’s income has no significant effect on the neighborhood taste-based mechanism, the income of the applicant has no impact on the perceived fit of the applicant in the neighborhood (b = 0.024, p>0.05), whereas ethnicity has (b = -0.227, p<0.001). Additionally, the unsignificant interaction effect of ethnicity, price and income show that neighborhood taste-based discrimination does not vary according to rental price and income, unlike owner taste-based discrimination. When we add the interaction between ethnicity and the question related to the scrappy neighborhood: *We want to rent out the apartment quick*, *it is a bit shabby and is located in a rough neighborhood*, *that’s why it is so cheap*, we find a positive significant effect on the neighborhood taste-based mechanism (b = 1.797, p<0.001). This result implies that ethnic minority candidates are steered towards poor quality dwellings and neighborhoods. A result that is in line with neighborhood taste-based discrimination. We should note however that these requests reveal negative information about the neighborhood by discussing bad experiences. As a result, any negative inferences about the candidates could match negative inferences about the neighborhood and could therefore interfere or wash out.

When we compare the results with the statistical based mechanisms in Table 6, we find further support for the theoretical separation of the different mechanisms of discrimination. First, the ethnic origin of the rental candidate has, among our population of students, no significant effect on the statistical mechanisms, which implies that ethnicity has no effect on the perceived financial reliability and communicational strength of the applicant. This result contradicts the theoretical rational of statistical discrimination in which the judgement of the agent on these characteristics is based on perceived ethnic group characteristics. Second, income has the biggest impact on the statistical mechanism (β = 0.637, p<0.001) and the price of the dwelling has, in contrast to the agent taste-based mechanism, a significant negative effect (b = -0.113, p<0.001).

However, we find no significant effect of the interaction variables in the analysis which implies that the statistical discrimination mechanism is absent or not fully measured in our vignette experiment. The agent seems to take the reported income as face value and is not giving it less weight based on priors concerning minority population. Finally, the discriminatory questions have little effect on the perceived financial reliability and communicational strength of the applicant.

Finally, we address effects of the candidate and client characteristics on the chance to be invited for a viewing in Table 7. These results provide us with the measures of discrimination that is also used in experimental fieldwork (audit studies, correspondence tests), namely the difference in chances of being invited to a dwelling viewing. First, ethnic minority candidates are significantly less invited to a viewing than their ethnic majority counter candidates (b = -0.625, p<0.001). Surprisingly, apart from the owner taste based mechanism, the effect of ethnicity is largest with regard to invitation rates (β = -0.222, p<0.001). Additionally, as with the other mechanisms, we find a positive effect from income on the invitation rate (b = 0.428, p<0.001). However, the interaction effect with ethnicity and income in model 2 shows that this positive effect does not hold for the ethnic minority group (b = -0.081, p<0.001). Second, for the owner’s preferences we find that for the neighbor’s request, the significant effect (b = -0.301, p<0.001) loses its significance when the interaction term with ethnicity (b = -0.392, p<0.01) is included in model 3. Which implies that a discriminatory request from a neighbor only affects the ethnic minority candidate’s chance to be invited. Contrastingly, when a “bad” neighborhood is mentioned, the not significant overall effect turns, when interacted with ethnicity, negative significant for the ethnic majority and positive significant for the ethnic minority candidate. More concretely, ethnic minority candidates have a higher chance to be invited when the dwelling “is a bit shabby and is located in a rough neighborhood”, whereas ethnic majority candidates have a lower chance to be invited for the same dwelling. This result clearly shows a *steering* mechanism, in which ethnic minority candidates are steered towards poor quality dwellings and neighborhoods [49]. Finally, for the last owner’s preference, the personal request for discrimination, we find that the general negative effect (b = -403, p<0.001) only holds for the ethnic minority candidate (b = -0.627, p<0.001) as shown in model 5. Compared to the other owner’s preferences, the personal request has most effect on the invitation rates (β = -0.158, p<0.001).

## 5. Conclusion and discussion

Whereas the “what”, “where” and “when” of ethnic discrimination are well-documented phenomena, there remains a big gap in addressing the “why” and “how” of racial discrimination [50]. Several authors make a plea for ‘mechanism-oriented’ analyses of discrimination and its origins by highlighting the multiple processes of housing discrimination that contribute to inequitable outcomes [51–53]. In this study we addressed this gap by theoretically differentiating between four mechanisms of rental discrimination: agent-, owner- and neighborhood taste-based discrimination and statistical discrimination. In addition, we introduced a new method for measuring these mechanisms of discrimination on the housing market, namely a multi factorial survey experiment. To the best of our knowledge, it is the first time that this technique has been used to investigated discrimination on the housing market. We introduced this new experiment among 576 students in real estate and related domains across different tertiary educational institutions in Belgium. Within the confines of this research population, we could draw the following conclusions.

Firstly, our method allows scholars to disentangle different mechanisms of discrimination presented in our theoretical framework. The good to perfect fit of our structural equation model and the regression analyses provided suggestive evidence that our vignette experiment is a good measure of at least three mechanisms of discrimination: agent-, owner- and neighborhood taste-based discrimination. The mechanism of statistical discrimination could, however, be less grasped by our experiment. More concretely, the perceived financial reliability and communication skills of the applicant have no significant effect on invitation rates and do not differ between Belgian and North-African applicants. Statistical discrimination involves taking group characteristics of ethnic groups into account, which is maybe too abstract for unexperienced, students. While real estate agents might treat self-reported income with more skepticism and thus rely more on general information, students might convey the same signal as true information. Additionally, we chose to use financial reliability as the signal for statistical discrimination and not the more direct form of statistical discrimination that is signaled through financial strength. Future research should include a condition where the candidate’s income is not shown, which would be a more accurate proxy for statistical discrimination.

Secondly, when comparing the four theoretical mechanisms it appears that the agent taste-based mechanism has the strongest effect on the invitation rates among students in real estate and related subjects. In other words, the personal preference for an applicant is the main driver for inviting applicants for a house visit. Although the literature on the dominant mechanism is inconclusive [54], Ahmed [31] found similar results in the field. Moreover, this agent taste-based mechanism shows pronounced ethnic inequalities, which suggests agent taste-based discrimination. In line with the theory, this agent taste-based discrimination does not vary according to the rental price, the income level of the applicant or particular (discriminatory) requests of clients.

Our results show that ethnic minority candidates have a significant decreased chance of invitation, in all our models, across almost all the mechanisms. As for the mechanisms that drive this inequality, we found that ethnic minority candidates are favored when the dwelling and the neighborhood are of poor quality. Even more, the ethnic majority candidate loses chances of invitation in this context. This result is in line with previous research in Belgium [55], and shows a clear presence of neighborhood taste based discrimination, in which the participant steers the ethnic minority candidates towards poorer quality dwellings and neighborhoods. For the owners that request to not rent out to ethnic minority candidates, we found that the participants not only adhere to the wishes of the client, but that this also results in actual discrimination in invitation rates for the ethnic minority group. Same result was found when the question was asked by the neighbor, however significantly smaller. These results indicate a clear presence of the owner taste based discrimination, which is in line with previous findings among Belgian realtors [15].

This study innovated in being the first to study the empirical relevance of the dominant theoretical mechanisms of ethnic discrimination on the housing market. However, fundamental to its setup and design, this study has several limitations. First, fundamental to a multifactorial survey experiment design is the laboratory setting it is situated in. Even though some studies found that these vignette experiments are good proxies for real life decision making and correlate highly with actual behavior, it is difficult to make claims concerning situations in the field [56]. We could not account for the presence of socially desirable answers, which may have translated in an overestimation of positive evaluations of ethnic minority candidates. Therefore, we expect especially the agent taste-based mechanism to be underestimated in our sample.

Second, inherent to testing the vignette experiments with pre graduate students is the weak ecological validity of our results. However, many studies on the labor market found that the assessment of job candidates did not differ substantially between professional recruiters and students [57]. Yet, we expect that our results underestimate the discriminatory behavior in the field. Primarily because when testing with students there is no job or costs of discriminating at stake, secondary because we found that the statistical mechanism was not supported in our data. We expect that professionals are more aware of the legal selection criteria when judging a rental candidate, which includes solvability, at least more aware than students in real estate or other domains and therefore, the statistical discrimination mechanism could be higher when tested with real estate agents in the field. An additional concern when working with students is the possibility of measuring signals about the unobservable of the respondent rather than their actual behavior. For example, all discriminatory requests had a negative effect on the agent taste based mechanism. These requests describe bad experiences, something that tends to happen in bad locations. While the reference category, asking for clean and quit renters, implies that the dwelling is situated in a nice neighborhood. The college student might be likely to see themselves identifying with the person who would move into a nice neighborhood, hence the same, negative effect for all request.

For these reasons, this study should be used as a starting point for future research on mechanisms of discrimination. Nevertheless, within the confines of these limitations, we introduced a new method to measure mechanisms of discrimination on the housing market and found strong evidence for the theoretical mechanisms of discrimination in our sample. Further research should include this broader view on mechanisms to add to the understanding of drivers of discrimination. Moreover, the combination of theoretical testing of dominant mechanisms in relation to actual discriminatory behavior tested with correspondence tests should yield results that could strengthen the literature on mechanisms of discrimination and result in a better understanding and approach to tackle ethnic discrimination on the housing market.

## Supporting information

S1 Data (CSV)

S1 Fig (TIF)

S2 Fig (TIF)
