# Supplementary figures and images for: Introducing a vignette experiment to study mechanisms of ethnic discrimination on the housing market
Source: PLoS One. 2022 Oct 27;17(10):e0276698. doi: 10.1371/journal.pone.0276698 (PMC9612456; doi:10.1371/journal.pone.0276698)

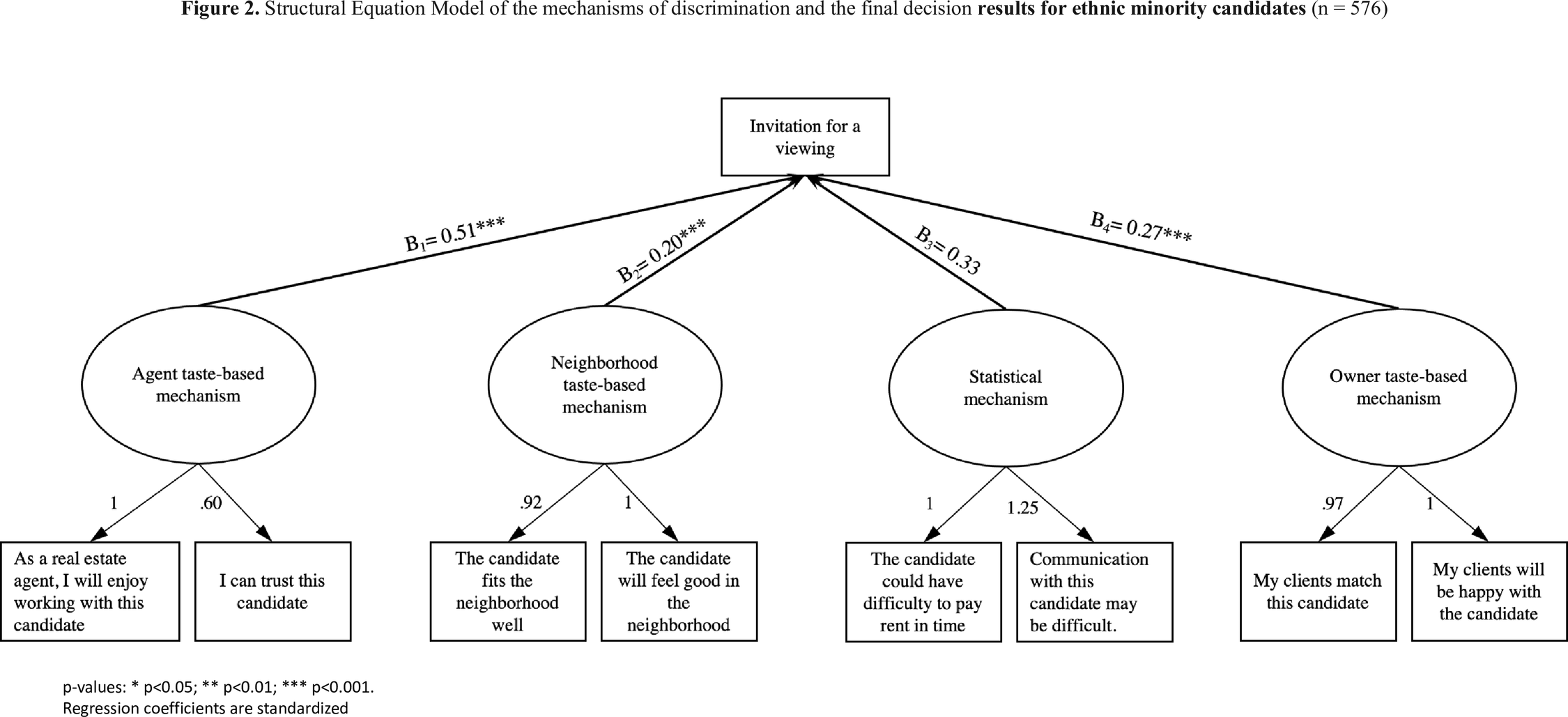

Supplement: S1 Fig — (TIF) [file pone.0276698.s002.tif]

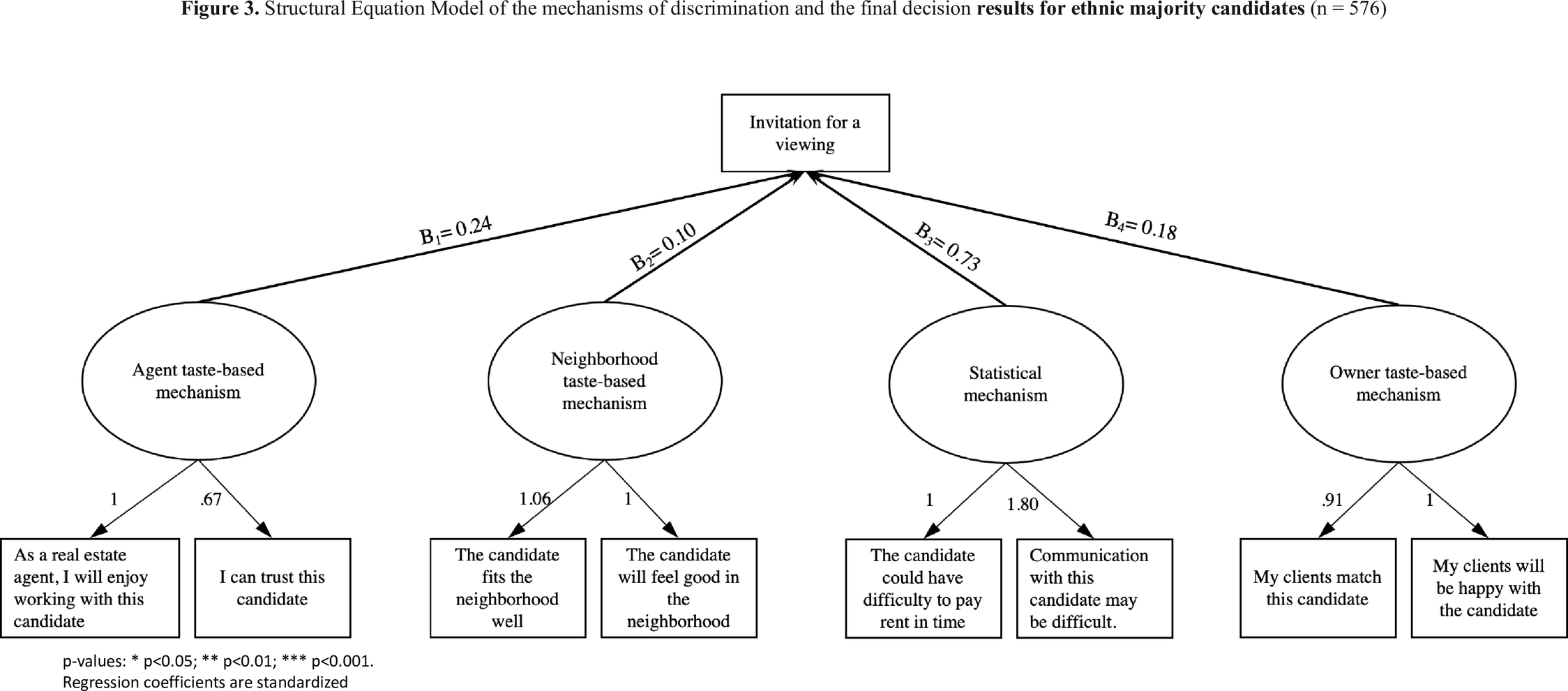

Supplement: S2 Fig — (TIF) [file pone.0276698.s003.tif]
